# The Involvement of Oral Pathogenic Bacteria, *Fusobacterium nucleatum* Subspecies *animalis* in the Pathogenesis of Human Esophageal Adenocarcinoma

**DOI:** 10.1016/j.gastha.2025.100660

**Published:** 2025-03-24

**Authors:** Taku Kitano, Shintaro Okumura, Tomonori Matsumoto, Shigeru Tsunoda, Tatsuto Nishigori, Kazutaka Obama, Eiji Hara

**Affiliations:** 1 Department of Surgery, Graduate School of Medicine, Kyoto University, Kyoto, Japan; 2 Department of Molecular Biology, Research Institute for Microbial Diseases, Osaka University, Suita, Japan; 3 Laboratory of Ploidy Pathology, Graduate School of Frontier Biosciences, Osaka University, Suita, Japan

**Keywords:** Esophageal Adenocarcinoma (EAC), Oral Pathogenic Bacteria, *Fusobacterium nucleatum* Subspecies *animalis*, DNA Damage, Reactive Oxygen Species (ROS)

## Abstract

**Background and Aims:**

While esophageal microbiota is known to be altered in patients with esophageal adenocarcinoma (EAC), specific bacterial species contributing to the pathogenesis remain unclear. We aimed to identify bacteria associated with the pathogenesis of EAC and to investigate their potential role.

**Methods:**

Microbiota in EAC tissues was explored by 16S-rRNA sequencing. The impact of intratumoral bacteria on tumor microenvironment was histologically evaluated. The influence by pathological bacteria on cancer cells was examined by the treatment of OE33 cell line with cultural supernatant of bacteria.

**Results:**

Oral pathogenic bacteria previously reported to increase in colorectal cancer patients were detected in 7 out of 31 EAC patients and were associated with poor prognosis. Intratumoral bacterial infiltration was significantly accompanied with local neutrophil infiltration and nuclear positivity of NF-κB in cancer cells. Furthermore, the culture supernatant of *Fusobacterium nucleatum* applied to OE33 cells led to an upregulation of proinflammatory cytokine and chemokine genes that facilitate neutrophil migration. Notably, most of the *F. nucleatum* subspecies (spp.) present in the EAC tissues were identified as spp. *animalis*. The culture supernatant of spp. *animalis* induced significantly more reactive oxygen species–mediated DNA damage in EAC cells than spp. *nucleatum*, the most representative spp. of *F. nucleatum*, indicating the significance of spp. *animalis* rather than spp. *nucleatum* in the pathogenesis of EAC.

**Conclusion:**

Intratumoral microbiota, especially the infiltration of oral pathogenic bacteria such as *F. nucleatum*, has a profound impact on the tumor microenvironment of EAC. Spp. *animalis* was predominant among *F. nucleatum* in the EAC tissues and induced significant DNA damage in cancer cells.

## Graphical abstract

**Figure:**
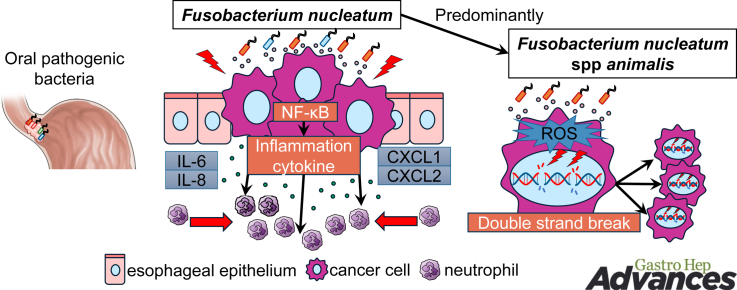

## Introduction

The incidence of esophageal adenocarcinoma (EAC) has been increasing throughout the world.^1^^,^^2^ Despite the development of intensive therapeutic strategies, the prognosis of EAC remains poor.^3^ Obesity, tobacco smoking and gastroesophageal reflux are epidemiologically proven as risk factors for the development of EAC.^4^ In addition, Barrett’s esophagus (BE), a metaplastic columnar epithelium in the esophagus is well known as the precursor to EAC. Some genomic alterations including mutations of TP53, CDKN2A, and chromosomal instability were also reported to be causally related to the development of EAC.^5^, ^6^, ^7^ However, the mechanisms of EAC development and progression remain largely unclear, and elucidating the pathophysiology of EAC has great potential significance to improve the long-term survival of patients with EAC.

In recent years, bacterial infections have been shown to be closely associated with tumor pathogenesis and prognosis. Intratumoral bacterial invasion in gastroenterological cancers has been reported to impact the characteristics of tumor cells, antitumor immunity, and the prognosis of cancer.^8^, ^9^, ^10^ Our previous investigation on gut microbiota in patients with colorectal cancer (CRC) identified 12 oral pathogenic bacterial species increasing in CRC patients, and revealed that 2 bacterial species, *Porphyromonas asaccharolytica* and *Porphyromonas gingivalis*, possessed a potential to promote CRC tumorigenesis.^11^ Human EAC tissues were also recently reported to exhibit intratumoral microbiota infection, the composition of which was different from that of normal esophageal mucosa.^12^, ^13^, ^14^ Given these findings, the pathogenesis and prognosis of EAC may also be affected by intratumoral microbiota, although it has not been examined in detail to date.

*Fusobacterium nucleatum* is one of the most examined oral pathogenic bacterial species for the relevance of gastroenterological cancers, especially CRC. Indeed, *F*. *nucleatum* was previously reported to have an impact on tumor development, metastasis and chemoresistance of CRC.^15^, ^16^, ^17^, ^18^ Although most of the previous studies demonstrated the pathological implications of *F*. *nucleatum* by in-vitro and in-vivo experiments using *F*. *nucleatum* subspecies (spp.) *nucleatum*,^16^^,^^18^, ^19^, ^20^ another spp. of *F*. *nucleatum*, spp. *animalis* was recently shown as the major *F*. *nucleatum* spp. existing in the CRC tissue.^21^, ^22^, ^23^, ^24^, ^25^ However, the impact of *F*. *nucleatum* spp. *animalis* on cancer has been scarcely studied, and little is known about the difference between *F*. *nucleatum* spp. *animalis* and spp. *nucleatum*.

In this study, we explored the microbial composition in EAC tissues with meta-16S analysis. Some of the 12 CRC-related oral pathogenic bacterial species were identified in the EAC tissue, and patients with those pathogenic bacteria had significantly poorer prognosis than their counterparts. Histological analyses also showed the association between the intratumoral infection of those oral pathogenic bacteria and alterations in immune microenvironment. We also demonstrated that *F*. *nucleatum* spp. *animalis* is the most predominant among the oral pathogenic bacterial species in the EAC tissues and has a potential to provoke genomic stress in EAC cells. Oral pathogenic bacteria, particularly *F*. *nucleatum* spp. *animalis*, would be an important target for exploring novel aspects of EAC pathogenesis.

## Materials and Methods

### Clinical EAC Specimens

The analysis included a total of 31 patients who underwent resection at Kyoto University Hospital between November 2005 and October 2022, without having received any preoperative anticancer therapy. The study protocol was reviewed and approved by the Ethics Committee of Kyoto University Hospitals (Kyoto University: Study No. R3873). Informed consent was waived due to the retrospective study design and the study information was disclosed on our hospital website, allowing eligible patients to opt out.

### DNA Extraction

DNA was extracted from 2 to 4 formalin-fixed paraffin-embedded (FFPE) slides of surgical specimens, including esophageal tumoral and nontumoral sections (10 μm), using the QIAamp DNA FFPE Tissue Kit (Qiagen, Hilden, Germany) following the manufacturer’s protocol. The extracted DNA was stored at −20 °C until further analysis.

### 16S-rRNA Gene Metasequencing and Analysis

Sequencing analysis of the V1-V2 region of bacterial 16S-rRNA gene was performed as described previously.^10^ Briefly, the V1-V2 target region was amplified by polymerase chain reaction (PCR) using primers shown in Table A1 and the KAPA HiFi Hot Start Ready Mix (Roche, Basel, Switzerland). The sequencing library was prepared from purified PCR amplicons, following the 16S library preparation protocol by Illumina. Indexes from Illumina were added to the 16S-PCR products to prepare the library. After confirming the quality of the library, 250 bp paired-end sequencing was performed using the MiSeq Reagent Kit v2 (500 cycles). The human sequence reads were removed from the sequencing data by mapping to the human reference genome (hg19) using Visual Studio Code.

The QIIME2 (version 2023.07) pipeline was used to process sequencing reads. Amplicon sequence variants (ASVs) were counted from denoised Fastq files using DADA2 plugin. The ASV counts were converted to the relative abundance per sample. A Naive Bayes classifier in the QIIME2 pipeline, trained on the SILVA138 database, was employed for phylogenetic classification with the representative sequences clustered at 99% similarity among detected ASVs. Identification of the specific bacterial species and spp. corresponding to each ASVs was performed by utilizing NCBI's updated (2024/01) 16S rRNA database and BLAST+ (v.2.15.0) for similarity searches.

### In Situ Hybridization (ISH)

In situ hybridization (ISH) targeting bacterial 16S-rRNA and 23S-rRNA of *F. nucleatum* in EAC tissues was performed using RNAscope® 2.5 HD Reagent Kit- RED (Advanced Cell Diagnostics (ACD), Newark, CA, USA, 322350) and Fast Red according to the manufacturer’s protocol. Briefly, FFPE sections were baked in an oven at 60 °C for 1 hour, deparaffinized, and then treated with RNA scope Hydrogen Peroxide for 10 minutes at room temperature. Antigen retrieval was performed by incubating the sections for 15 minutes in Target Retrieval Reagent preheated to 98 °C, followed by protease treatment with RNA scope Protease Plus at 40 °C for 30 minutes. Each probe in the kit was sequentially applied at the appropriate temperature and time according to the protocol. After signal development with Fast Red, nuclear counterstaining was performed using hematoxylin, followed by treatment with 0.02% ammonia water, and the sections were then mounted.

The probes were as follows: RNAscope® Probe-EB-16S rRNA (ACD, 464461) targeting bacterial 16S-rRNA (350-835 of NR_075051.2)^10^ and RNAscope® Probe-B-Fusobacterium-23S-3zz (ACD, 486411) targeting *F. nucleatum* (539204 - 539365 of CP003723).^20^

### Sanger Sequence Analysis of *F. nucleatum* spp.

PCR targeting the *nusG* gene (region 2: 2240390–2240454 of CP084163.1) was performed on 3 cases positive for *F. nucleatum* in both ISH and 16S-sequencing. Nested PCR was performed using primers shown in Table A1 and KOD FX Neo (TOYOBO, Osaka, Japan, KFX-201). The *nusG* region 2 primer was designed by identifying a relatively distinct sequence between spp. *nucleatum* and spp. *animalis* in the NCBI database (region 2: 80% similarity between *F. nucleatum* spp. *nucleatum* and spp. *animalis*).

PCR products were purified using NucleoSpin Gel and PCR clean-up (MACHEREY-NAGEL, Düren, Germany, 740609.250), and sequenced with 3500xL Genetic Analyzer (Thermo Fisher Scientific, 4405633).

### Immunohistochemistry (IHC)

For immunostaining, FFPE sections (5 μm) were deparaffinized, rehydrated, and treated with heat-induced epitope retrieval with Antigen Retrieval Buffer (100X Citrate Buffer) (Abcam, Cambridge, UK, ab93678) in a pressure cooker for IHC. The following antibodies were used: CD8 (no dilution, Nichirei Bioscience, Tokyo, Japan, 413201), CD66b (1:50, BioLegend, San Diego, CA, USA, 305102), NF-κβ p65 (1:800, Cell Signaling Technology, Danvers, MA, USA, 8242), and secondary antibodies including antirabbit (Vector Laboratories, Burlingame, CA, MP-7401), antimouse (Vector Laboratories, MP-7402). All slides were stained with their primary antibody at 4 °C overnight. The nuclei were counterstained with hematoxylin.

### Cell Culture

The EAC cell line OE33 was obtained from AddexBio (Shanghai, China). Cells were maintained in Roswell Park Memorial Institute 1640 medium (Nacalai Tesque, Kyoto, Japan) supplemented with 10% fetal bovine serum (Biowest, Nuaillé, France) and 5% penicillin-streptomycin. In each experiment, the culture supernatant of the bacteria was administered to the cells at a 100-fold dilution, N-Acetyl-L-cysteine (Nacalai Tesque, 00512-42) was administered at 5 mM with bacterial supernatant, and the administration period was 3 days.

### Bacteria Culture

*F. nucleatum* subsp. *animalis* JCM 11025, *F. nucleatum* subsp. *nucleatum* JCM 8532, *P. gingivalis* JCM 8525 and *Prevotella intermedia* JCM 11150 were obtained from the RIKEN BRC through the National Bio-Resource Project of the MEXT, Japan. The bacteria were anaerobically cultured with Gifu Anaerobic Medium (Nissui Pharmaceutical Co, Ltd, Tokyo, Japan, 05422). For the preparation of bacterial conditioned medium, each bacterial strain was cultured until reaching the stationary phase. The cultured medium was collected and centrifuged at 10,000 × g for 10 min. Following filtration through a 0.22-μm filter, the conditioned medium was collected and stocked at −80 °C until use.

### Quantitative Real-Time PCR

Total RNA was extracted using TRIzol (Thermo Fisher Scientific, Waltham, MA, USA, 15596018) and cDNA was synthesized using a PrimeScript RT reagent Kit with gDNA Eraser (Takara Bio Inc, Shiga, Japan, RR047A) according to the manufacturer’s protocol. Quantitative real-time PCR was performed on a Thermal Cycler Dice Real-Time System III (Takara Bio) using TB Green Premix Ex Taq II (Takara Bio, RR820A). The mRNA expression levels of each gene were calculated relative to β-actin expression levels. Primers used are shown in Table A1.

### Immunofluorescence Staining

Treated cells were fixed in 4% paraformaldehyde and subjected to immunofluorescence staining using primary and secondary antibodies including mouse antiphospho-Histone H2A.X (Ser139) (1:1000, Sigma-Aldrich, St. Louis, MO, USA, 05-636), rabbit anti-53BP1 (1:500, Novus Biologicals, Littleton, CO, USA, NB100-304), Alexa Fluor Plus 488-conjugated antirabbit (1:1000, Thermo Fisher Scientific, A32790) and Alexa Fluor Plus 555-conjugated antimouse (1:1000, Thermo Fisher Scientific, A32773). The nuclei were counterstained with 4',6-diamidino-2-phenylindole (1:1000, Thermo Fisher Scientific, F10347). Fluorescence images were analyzed using a fluorescence microscope (BZ-X710; Keyence, Osaka, Japan) and BZ-X analyzer software (Keyence).

### Neutral Comet Assay

The treated cells were collected and resuspended in phosphate buffered saline (PBS) to 3–5×10^5^ cells/ml. One percent low-melting agarose (Lonza, Basel, Switzerland, 50100) was added to the cell suspension at a 3:4 volume ratio. The mixture was dropped onto slides precoated with low-melting agarose gel, followed by incubation with lysis solution (Bio-Techne, Minneapolis, MN, USA, 4250-050-01) for 30 minutes at 4 °C. After rinsing the lysis solution with TBE buffer, neutral electrophoresis was performed for 20 minutes at 30 volts in TBE buffer, and the slides were rinsed with 70% ethanol for 5 minutes and air-dried. After staining DNA with Midori Green Direct (Nippon Genetics, Tokyo, Japan, NE-MG06), images were captured using a BZ-X710 fluorescence microscope (Keyence). The olive tail moment, defined as the percentage of DNA in the tail multiplied by the tail moment length, was determined utilizing CometScore 2.0 software. In all experiments, the olive tail moment was determined utilizing 50 comet tails.

### ROS Assay

CellROX Deep Red Reagent (Thermo Fisher Scientific, C10422) at a final concentration of 5 μM was added to the cells treated with condition medium and incubated for 30 minutes at 37 °C. After fixation in 4% paraformaldehyde, the nuclei were counterstained with 4',6-diamidino-2-phenylindole, and fluorescence images were analyzed using a fluorescence microscope and ImageJ.

### Statistical Analysis

Statistical analyses were performed using the Mann–Whitney U test, Student’s t-test, Fisher's exact test, log-rank test or Cox proportional hazards model using RStudio software (version 2023.06.1 + 524) and Microsoft Office Excel (Microsoft).

## Results

### Oral Pathogenic Bacteria Were Identified in the Tumor Tissue of Esophageal and Esophagogastric Junction (EGJ) Adenocarcinoma

**Figure 1:**
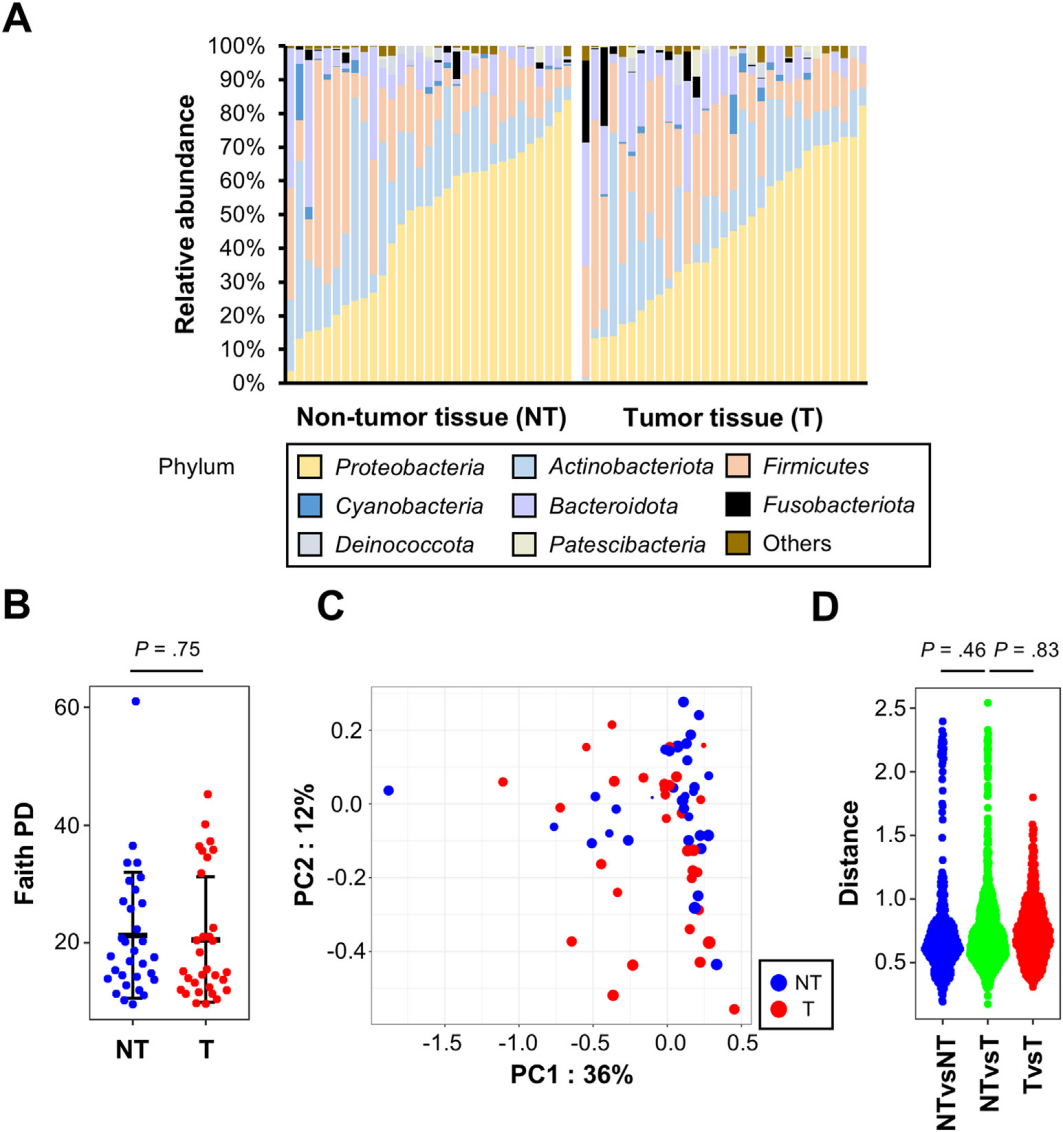
16S metagenomic sequencing of human EAC. (A) Taxonomic profiles of predominant bacterial phylum in EAC and nontumoral tissues (n = 31 in each group). Relative abundance is shown. (B) Comparative analysis of the alpha diversity. Faith’s phylogenetic diversity index was used. Error bars indicate mean ± standard deviation *P* value was calculated by t-test. (C) Principal coordinate analysis using weighted-UniFrac distance of beta diversity between EAC and nontumoral tissues. (D) UniFrac distance comparison between EAC and nontumoral tissues. *P* value was calculated by PERMANOVA-test.

To investigate the microbial involvement in EAC, we examined the bacterial composition of the tumor tissues and the nontumor tissues in 31 EAC patients who underwent subtotal esophagectomy without preoperative therapy. Meta-16S rRNA gene sequencing identified bacterial entity in both tissue types, and their composition was grossly comparable between the tumors and nontumors (Figure 1A). Analysis on the diversities also detected no significant difference in the microbial composition between the 2 groups (Figure 1B–D).

**Figure 2:**
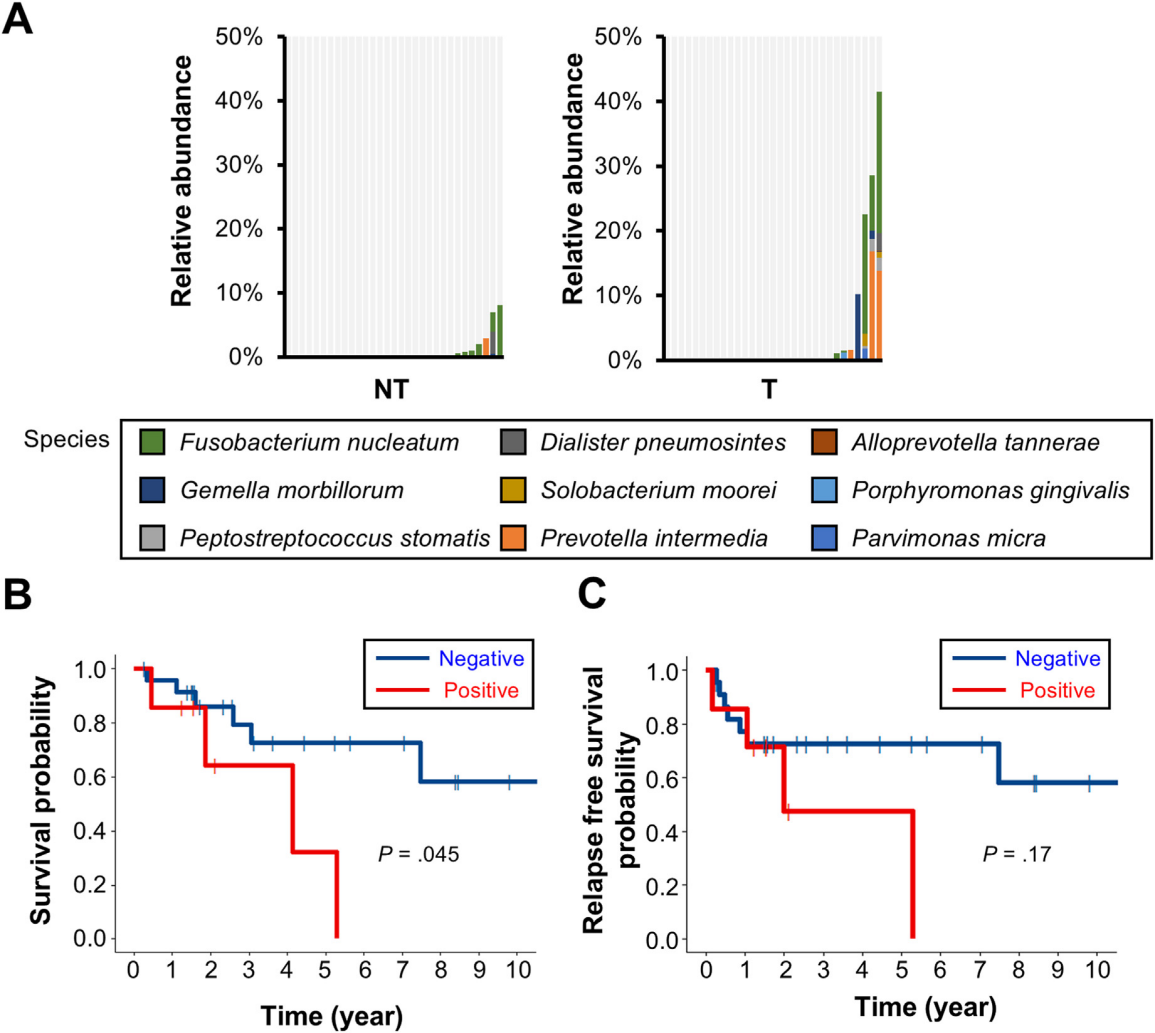
Profiles of oral pathogenic bacteria and survival outcome. (A) The species of oral pathogenic bacteria increased in colorectal cancer patients based on 16S metagenomic sequencing in EAC and nontumoral tissues. Relative abundance is shown. (B-C) Kaplan–Meier curves for overall survival (B) and relapse-free survival (C) of EAC patients with and without oral pathogenic bacteria. *P* value was calculated by log-rank test.

**Figure A1:**
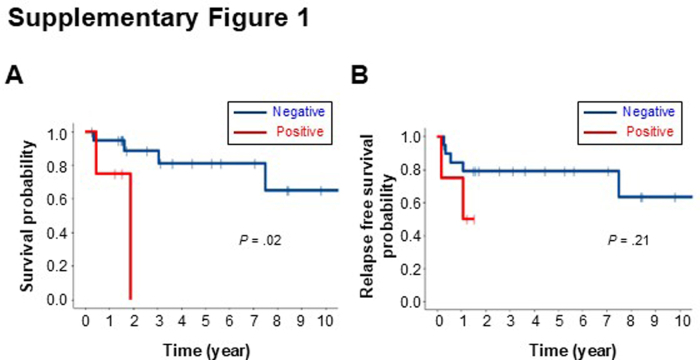
Survival outcome of EAC patients younger than 80 years. (A-B) Kaplan-Meier curves for overall survival (A) and relapse-free survival (B) of EAC patients younger than 80 years with and without oral pathogenic bacteria. *P* value was calculated by Log-rank test.

Interestingly, we found that the 12 oral pathogenic bacterial species, which were dominantly identified in CRC patients in our previous study,^11^ were also frequently detected in EAC tissues (7/31, 22.6%, Figure 2A). To investigate the clinical relevance of the intratumoral entity of these oral pathogenic bacteria, clinical data of the 31 patients were analyzed. The age composition of the 7 patients positive for the oral pathogenic bacteria (the positive patients) was significantly older than that of the 24 patients negative for the oral pathogenic bacteria (the negative patients). There were no significant differences between the 2 groups in the sex ratio, the distribution of body mass index, the proportion of smokers, and the use of proton pump inhibitors, each of which potentially affects microbial composition in the gastrointestinal tract.^26^, ^27^, ^28^, ^29^ Notably, while there was no significant difference in the profiles of histological differentiation, tumor diameter or tumor stage according to the Union for International Cancer Control Tumor, Node, and Metastasis classification between the 2 groups (Table), overall survival of the positive patients were significantly poorer than that of the negative patients (Figure 2B). Relapse-free survival of the positive patients also tended to be poorer than that of the negative patients, though the difference was not statistically significant (Figure 2C). This tendency was more clearly observed in the subpopulation younger than 80 years (Figure A1A and B, Table A2). Although the positivity of the oral pathogenic bacteria was not an independent prognostic factor in a multivariate analysis with a Cox proportional hazards model, its overall survival (OS) and relapse free survival (RFS) hazard ratios (HRs) were 1.51 (95% confidence interval [CI]: 0.28–8.24) and 1.38 (95% CI: 0.31–6.07), respectively (Table A3). These findings suggested that the 12 oral pathogenic bacteria might be related to the prognosis of EAC.

**Table:** Characteristics of the EAC Patients Enrolled in This Study

Characteristics	Negative (n = 24)	Positive (n = 7)	*P*-value
Age, y *mean*	68.6 (48–84)	77.9 (66–87)	.02
Sex, *N*			1
Female	3	1	
Male	21	6	
BMI, kg/m^2^*mean*	23.3 (17.2–36.9)	21.2 (18.6–23.8)	.22
Current smoker, *N*	5	1	1
PPI user, *N*	8	3	1
Tumor size, mm *mean*	52.5 (8–140)	54.4 (30–80)	.89
Histology, *N*			.57
pap, tub	15	3	
Por	4	2	
Endoscopic findings, *N*			
Reflux esophagitis	13	1	.09
Atrophic gastritis	5	2	.64
Barrett’s esophagus	12	0	.03
Location of tumor epicenter, *N*			.29
Within 2 cm from EGJ	18	7	
Above 2 cm from EGJ	6	0	
Completion of adjuvant chemotherapy, *N*	5	1	.587
TNM classification^a^, *N*			
T Classification			.48
T1	9	1	
T2	3	2	
T3	9	3	
T4	3	1	
N classification			.54
N0	5	2	
N1	12	1	
N2	2	2	
N3	5	2	
M Classification			.34
M0	23	6	
M1	1	1	
Stage			.66
Ⅰ	4	1	
Ⅱ	6	1	
Ⅲ	8	3	
Ⅳ	6	2	

Negative, patients negative for the oral pathogenic bacteria; Positive, patients positive for the oral pathogenic bacteria.

BMI, body mass index; EGJ, esophagogastric junction; n, number; PPI, proton pump inhibitor; TNM, Tumor, Node and Metastasis; UICC, Union for International Cancer Control.

a UICC, 8th edition.

### Invasion of Oral Pathogenic Bacteria into the Tumor Tissue Might Impact the Tumor Microenvironment and the Signal Activity of EAC Cells

**Figure 3:**
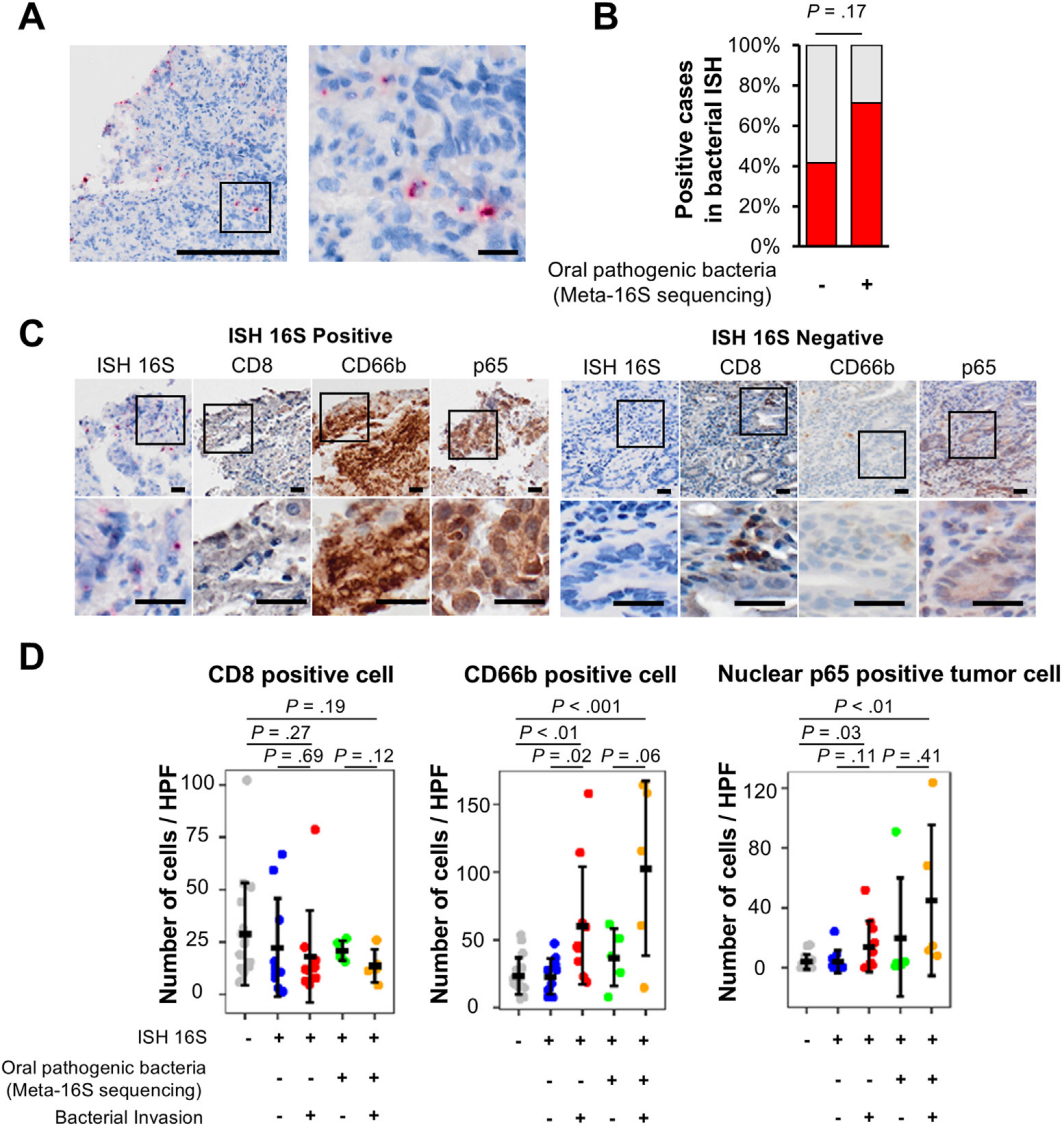
Relationship between intratumoral bacterial infection and immune cell infiltration. (A) Representative images of ISH targeting bacterial 16S-rRNA gene. Positive signals are indicated by red foci. Scale bars, 200 μm (left panel), 20 μm (right panel). (B) The positivity of 16S-rRNA-targeted ISH in cases with and without 12 oral pathogenic bacteria. (C) Representative images of immunohistochemistry for tumor-infiltrating CD8^+^ T cells, neutrophils, and NF-κβ in EAC tissues. Scale bars, 25 μm. (D) Numbers of CD8^+^ T cells, neutrophils, and NF-κβ positive tumor cells in EAC tissues. Immuno-stained images were evaluated in 5 groups based on the positivity of bacterial 16S-rRNA by ISH and the presence of the 12 oral pathogenic bacteria. In tumors positive for ISH of bacterial 16S-rRNA, areas around 16S-rRNA ISH signals and those without ISH signals were separately analyzed. Each group was evaluated with 3 randomly selected fields, and their averages are shown.

To investigate the impact of microbial invasion on the tumor microenvironment, we examined the spatial distribution of bacteria using ISH with a probe targeting all bacterial species.^10^ Stains detecting intratumoral bacterial infection was observed in 48.4% of EAC tissues. Notably, the bacterial entity was observed not only on the surface but also in deeper areas of the tissue (Figure 3A). The tumors positive for the 12 oral pathogenic bacteria (71.4%) were more likely to exhibit ISH stains compared to the others (41.7%) (Figure 3B). In addition, the accumulation of CD66b-positive cells was observed in the vicinity of the bacterial entity, while the migration of CD8-positive cells seemed to be negatively correlated with the bacterial invasion (Figure 3C and D). Interestingly, these histological features were clearer in the positive patients than the negative patients (Figure 3D), and the enhancement of nuclear translocation of NF-κβ (p65) in the tumor cells was observed accompanying these results (Figure 3C and D). In summary, it was suggested that the invasion of the oral pathogenic bacteria might have impact on the immune microenvironment in the tumor tissue of EAC and activate specific signal pathways in EAC cells.

### *F*. *nucleatum* spp. *animalis* was the Most Prevalent Oral Pathogenic Bacteria in the EAC Tissue

**Figure 4:**
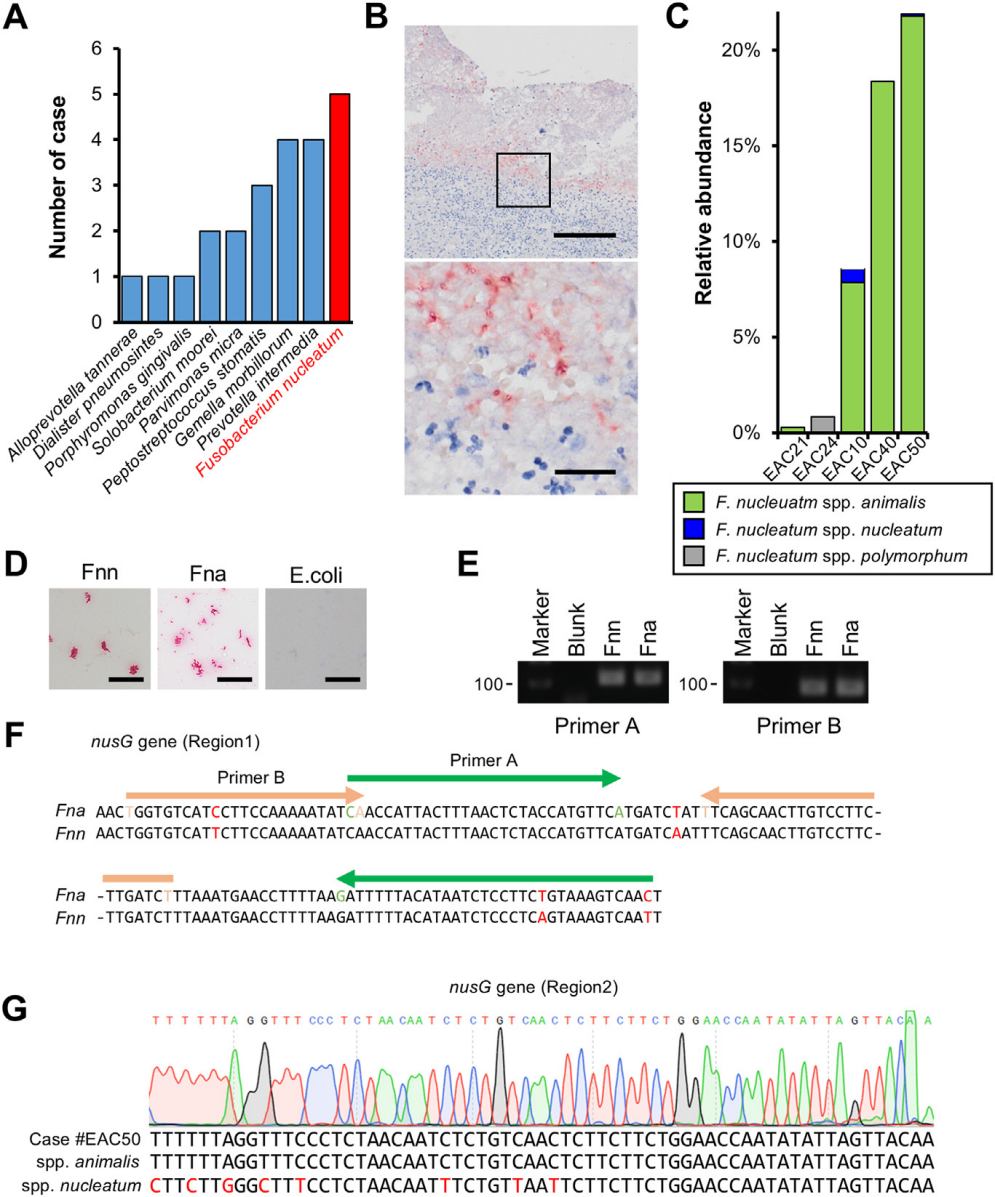
Subspecies analysis of *F. nucleatum* in tumor tissue. (A) The number of cases with intratumoral infection of 12 oral pathogenic bacteria. (B) Representative images of ISH targeting the 23S-rRNA gene of *F. nucleatum*. Scale bars, 25 μm. (C) *F. nucleatum* subspecies detected by 16S metagenomic sequencing. Relative abundance among total bacteria in each case is shown. (D) Representative images of ISH targeting the 23S-rRNA gene of *F. nucleatum* in *F. nucleatum* spp. *nucleatum*, *F. nucleatum* spp. *animalis* and *Escherichia coli*. Scale bars, 50 μm. (E) Agarose gel electrophoresis (3% agarose) of PCR-amplified products using 2 primer sets targeting *nusG* gene (region 1). (F) Sequence of *nusG* gene (region 1) of *F. nucleatum* spp. *nucleatum* (ATCC 25586), spp. *animalis* (KCOM 1325) and primer sets. (G) Representative sequencing data of *nusG* gene (Region 2) in the EAC tissue (#EAC50), *F. nucleatum* spp. *nucleatum* and spp. *animalis*.

Among the 12 oral pathogenic bacteria, *F*. *nucleatum* was most frequently detected in the EAC tissues, while *P*. *gingivalis* was detected only from one patient and *P*. *asaccharolytica* was not detected in any patients (Figure 4A). Although the relevance of *F*. *nucleatum* to EAC was not well researched, this bacterial species was reported to promote tumorigenesis and disease progression of CRC.^16^^,^^18^, ^19^, ^20^ In addition, a recent study reported that *F*. *nucleatum* also exist in the tumor tissue of esophagogastric junction (EGJ) and gastric adenocarcinoma and was related to the poor prognosis of the disease.^30^ Therefore, we focused on *F*. *nucleatum* to study the relationship between the intratumoral bacterial infection and the pathophysiology of EAC. First, ISH with a probe specific to *F*. *nucleatum*^20^ was carried out for all patients. Positive staining was observed in the tumor tissue of 7 patients (22.6%) including some of those negative for *F*. *nucleatum* with meta-16S sequencing (Figure 4B). This result possibly indicated that the intratumoral infection of *F*. *nucleatum* would be present more frequently than that could be expected with meta-16S sequencing.

*F*. *nucleatum* is a heterogeneous species, including 4 spp.: spp. *nucleatum*, spp. *vincentii*, spp. *polymorphum*, and spp. *animalis*. It has been proposed that these 4 spp. are sufficiently divergent to be classified as different species.^31^ Thereby, we tried to identify spp. of *F*. *nucleatum* existing in the EAC tissue. The detailed analysis of meta-16S sequencing revealed that most bacterial taxa belonging to *F*. *nucleatum* in the tumor tissue corresponded to *F*. *nucleatum* spp. *animalis* (Figure 4C). Both the quantitative PCR targeting a region of *nusG* gene (region 1) that was widely performed to detect *F*. *nucleatum* in clinical specimens^32^, ^33^, ^34^, ^35^ and *F*. *nucleatum*-specific ISH^20^ failed to distinguish spp. *animalis* and spp. *nucleatum*, the representative type strain of *F*. *nucleatum* (Figure 4D–F). Sequencing of a spp.-specific region of *nusG* gene (region 2) identified that *nusG* gene existing in the EAC tissues corresponded to that of *F*. *nucleatum* spp. *animalis*, but not spp. *nucleatum* (Figure 4G). These results suggested that the majority of *F*. *nucleatum* existing in the EAC tissues was *F*. *nucleatum* spp. *animalis*.

### *F*. *nucleatum* spp. *animalis* Provoke Oxidative Stress and DNA Damage in EAC Cells

**Figure 5:**
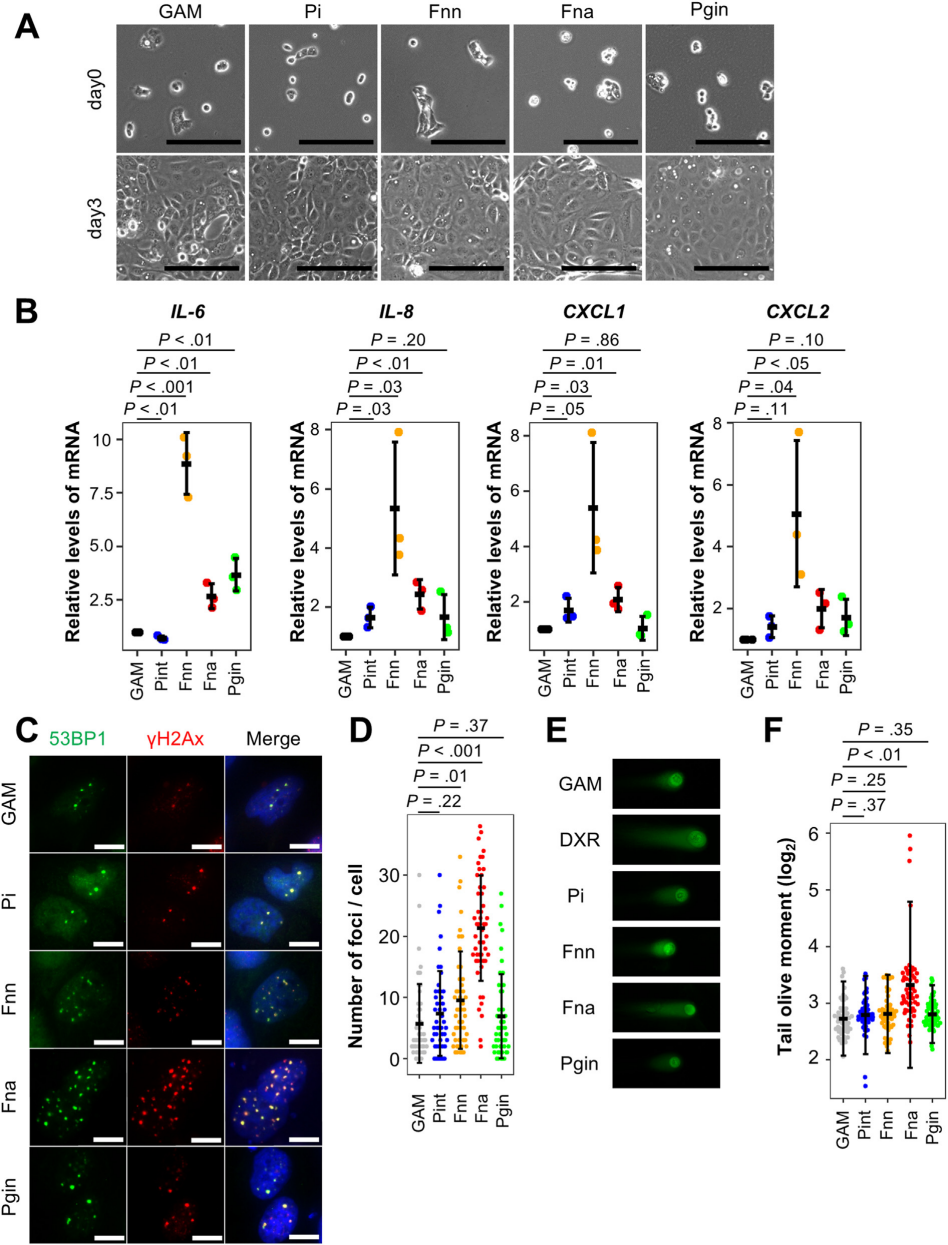
Influence of bacterial supernatant on EAC cells. (A) Representative images of EAC cells (OE33 cells) treated with each bacterial supernatant. *Prevotella intermedia* (Pi), *F*. *nucleatum* spp. *nucleatum* (Fnn), *F*. *nucleatum* spp. *animalis* (Fna) *P*. *gingivalis* (Pgin). Treatment with Gifu Anaerobic Medium was set as a negative control. (B) Relative expression of inflammatory cytokine or chemokine genes in EAC cells cultured with the bacterial conditioned media. β-actin was used as an internal control. (C-D) Immunofluorescence images of 53BP1 and γH2AX staining (C) and the number of γH2AX- and 53BP1-double positive foci per cell (D). Scale bar, 10 μm. (E-F) Representative images (E) and olive tail moment (F) in neutral comet assay. In (D) and (F), n = 50 in each group. In each assay, OE33 cells were treated with a 1/100 dilution of bacterial supernatant for 3 days, and treatment with Gifu Anaerobic Medium was set as a negative control. Data were obtained by 3 or more biological independent experiments. In all dot plots, error bars indicate mean ± standard deviation.

Whereas *F*. *nucleatum* spp. *nucleatum* was well studied for its oncogenic virulence, little was known about that of *F*. *nucleatum* spp. *animalis*. We therefore tried to identify the biological significance of the intratumoral infection of *F*. *nucleatum* spp. *animalis* for the pathophysiology of EAC. EAC cells (OE33 cells) were cultured in media containing bacterial culture supernatants of *Prevotella intermedia*, a second dominant oral pathogenic bacteria in the EAC tissue, *F*. *nucleatum* spp. *nucleatum*, *F*. *nucleatum* spp. *animalis*, and *P*. *gingivalis*. As a result, while the cell growth of EAC cells was not affected by any of the bacterial culture supernatants (Figure 5A), alterations of gene expressions of *IL-6*, *IL-8*, *CXCL1* and *CXCL2* were observed in EAC cells cultured with the bacterial conditioned media (Figure 5B). Although the gene expression levels were most significantly upregulated in cells cultured in a bacterial-conditioned medium of *F*. *nucleatum* spp. *nucleatum*, there was also a statistically significant increase in cells cultured in a bacterial-conditioned medium of *F*. *nucleatum* spp. *animalis*. On the contrary, the expressions of only some of the genes were elevated in cells cultured with bacterial conditioned media of *P*. *intermedia* and *P*. *gingivalis*. This result suggested that some of the oral pathogenic bacteria including *F*. *nucleatum* spp. *animalis* have an ability to stimulate EAC cells to secrete cytokines and chemokines that promote chemotaxis of neutrophiles.

We next examined DNA damage in cells cultured with each conditioned medium. Notably, nuclear coexistence of 53BP1 and γH2AX, and DNA double-strand breaks clearly increased in cells cultured with the bacterial conditioned medium of *F*. *nucleatum* spp. *animals*. In marked contrast, in cells cultured with the bacterial conditioned medium of *F*. *nucleatum* spp. *nucleatum*, the coexistence of 53BP1 and γH2AX was detected significantly less often than those cultured with spp. *animalis*, and the increase of DNA double-strand breaks was not evident (Figure 5C–F). Both coexistence of 53BP1 and γH2AX, and DNA double-strand breaks did not increase in cells cultured with bacterial conditioned media of *P*. *intermedia* and *P*. *gingivalis* (Figure 5C–F).

**Figure 6:**
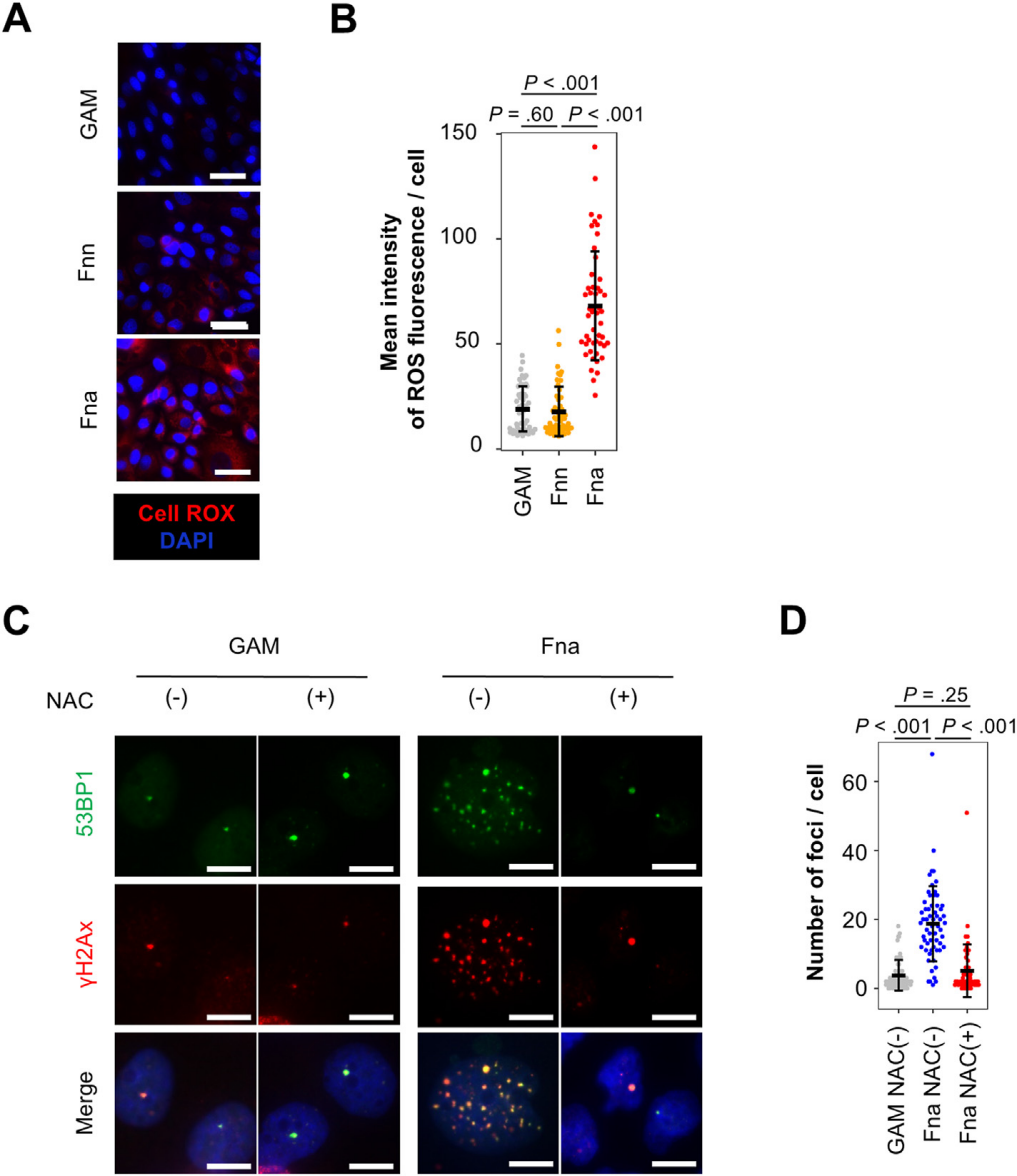
*Fusobacterium nucleatum* spp. *animalis* provokes DNA damage in EAC cells through ROS accumulation. (A-B) Detection of intracellular ROS in cells cultured with bacterial conditioned medium. *F*. *nucleatum* spp. *nucleatum* (Fnn), *F*. *nucleatum* spp. *animalis* (Fna). Treatment with GAM was set as a negative control. ROS was stained by the CellROX DeepRed reagent, and representative fluorescent microscopic images (A) and their mean fluorescence intensity per cell (B) are shown. Scale bar, 50 μm. (C-D) DNA damage foci in cells treated with and without N-Acetyl-L-cysteine. OE33 cells were treated with either GAM medium or supernatant of *F. nucleatum* spp. *animalis* (Fna) with and without 5 mM N-Acetyl-L-cysteine. Representative images (C) and the number of γH2AX- and 53BP1-double positive foci per cell (D) are shown. Scale bar, 10 μm. In (B), n = 50 in each group, and in (D), n = 60 in each group. In each assay, OE33 cells were treated with a 1/100 dilution of bacterial supernatant for 3 days, and treatment with GAM was set as a negative control. Data were obtained by 3 or more biological independent experiments. In all dot plots, error bars indicate mean ± standard deviation. GAM, Gifu Anaerobic Medium.

Furthermore, the accumulation of reactive oxygen species (ROS) significantly increased in cells cultured with the bacterial conditioned medium of *F*. *nucleatum* spp. *animalis*, but not with those of other bacterial species including *F*. *nucleatum* spp. *nucleatum* (Figure 6A and B). When N-acetyl cysteine that works as a radical scavenger was added in the culture medium, DNA damage induced by *F*. *nucleatum* spp. *animalis* was clearly canceled (Figure 6C and D). As a summary of these results, it was suggested that *F*. *nucleatum* spp. *animalis* have a distinctive potential to induce ROS accumulation and provoke DNA damage in the EAC cell.

**Figure A2:**
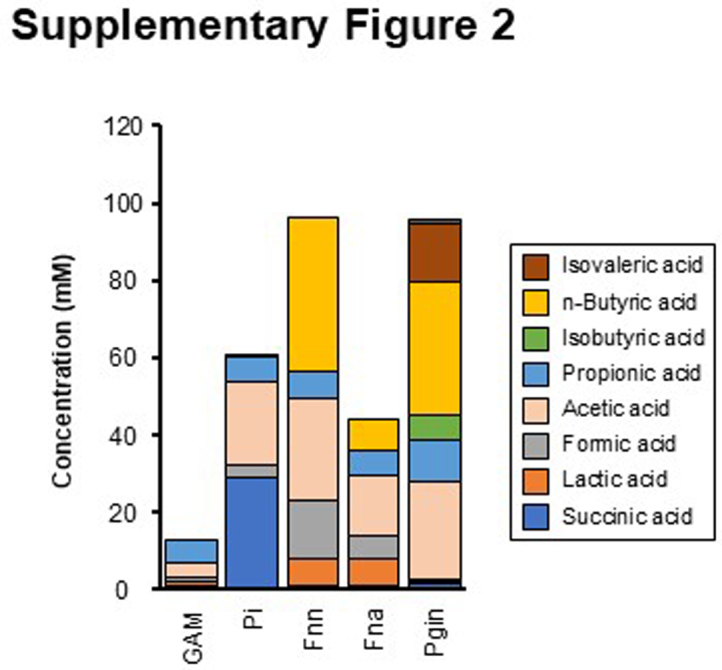
The concentration of the short chain fatty acids (SCFAs) in bacterial supernatant. Concentrations of SCFAs present in the indicated bacterial conditioned media or plain bacterial culture media of GAM (Mock) are shown. Data were obtained by 3 biological independent experiments.

In the previous study, we reported that *P. gingivalis* induced DNA damage response and promoted inflammatory gene expressions in cells via butyrate secretion.^11^ *F. nucleatum* is also known to be an oral pathogenic bacterium secreting butyrate.^36^ However, the butyrate concentration in the bacterial culture supernatant of *F. nucleatum* spp. *animalis* was only 8.3 mM, whereas that in the bacterial culture supernatant of *F. nucleatum* spp. *nucleatum* was 39.5 mM that was almost equal to that in the bacterial culture supernatant of *P. gingivalis* (34.0 mM) (Figure A2). Additionally, the bacterial culture supernatant was added to the cell culture medium at a ratio of 1/100 in the current study, while the ratio was 1/30 in our previous study.^11^ Therefore, the butyrate concentration in the bacterial conditioned medium of *F*. *nucleatum* spp. *animalis* did not seem enough to provoke DNA damage. Together, these data suggest that *F*. *nucleatum* spp. *animalis* secretes a yet unidentified virulence factor other than butyrate that induces oxidative stress and DNA damage in EAC cells.

## Discussion

In the current study, the species-level meta-16S rRNA gene sequencing analysis identified the infection of several oral pathogenic bacterial species such as *F*. *nucleatum* and *P*. *gingivalis* species in EAC tissues. The intratumoral infection of oral pathogenic bacteria has been recognized to have an impact on the progression of various cancers including CRC, gastric cancer, pancreatic cancer, and liver cancer.^37^ The intratumoral infection of *F*. *nucleatum* and *P*. *gingivalis* species has also been identified in esophageal squamous cell carcinoma^38^, ^39^, ^40^, ^41^, ^42^ and cancers arising in the stomach and EGJ.^30^ Along with these studies, our findings demonstrating the presence of *F*. *nucleatum* and *P*. *gingivalis* in EAC tissues suggest that these oral pathogenic bacteria are deeply involved in the pathogenesis of upper gastrointestinal cancers.

Interestingly, patients positive for oral pathogenic bacteria had significantly poorer prognosis compared to the negative patients. Whereas there were no significant differences in the oncological profiles between these 2 groups, the age composition in the positive patients was significantly older than that of the negative patients. We therefore additionally analyzed the prognosis in the subpopulation younger than 80 years to avoid the influence of the therapeutic restriction. The poor prognosis of the positive patients was more clearly observed, while the age composition was not significantly different between the 2 groups in the subpopulation. Because of the small sample size of the current study, the positivity of the oral pathogenic bacteria was not detected as an independent prognostic factor for both OS and RFS. A further study with a larger population should help clarify the significance of the positivity of the oral pathogenic bacteria for EAC prognosis. We showed that the accumulation of CD66b-positive cells and decrease in CD8-positive cells in the bacteria invasion area of the EAC tissue were prominent in the positive patients (Figure 3). Importantly, *F*. *nucleatum* and *P*. *gingivalis* have been reported to be associated with the suppression of immune defense against CRC.^19^^,^^43^^,^^44^ Given these findings, the intratumoral invasion of the oral pathogenic bacteria such as *F*. *nucleatum* and *P*. *gingivalis* may enhance the progression of EAC through the suppression of antitumor immunity. Although further research is needed to clarify the involvement of bacterial infections in the tumor immune microenvironment and tumor progression, these infections might become promising therapeutic targets to reinforce the therapeutic effect of immune-checkpoint agents against EAC.

*F*. *nucleatum* was most frequently detected among the oral pathogenic bacteria in the EAC tissues. Notably, we found that *F*. *nucleatum* in EAC was predominantly composed of spp. *animalis*, rather than the representative spp. *nucleatum* by the meta-16S rRNA gene sequencing and additional analysis of spp.-specific sequences in *nusG* gene (region 2). Unfortunately, neither quantitative PCR targeting region 1 of *nusG* gene^32^, ^33^, ^34^, ^35^^,^^38^^,^^39^ nor ISH^20^ that have widely been used to identify *F*. *nucleatum* in the clinical samples can distinguish spp. *animalis* from spp. *nucleatum*. Furthermore, since the representative type strain of *F*. *nucleatum* is *F*. *nucleatum* spp. *nucleatum* ATCC 25586 (VPI 4355),^45^ previous studies examining the pathological implications of *F*. *nucleatum* in cancer development mostly used a bacterial strain of *F*. *nucleatum* spp. *nucleatum* for experimental manipulation.^16^^,^^18^, ^19^, ^20^^,^^30^^,^^39^, ^40^, ^41^ Indeed, *F*. *nucleatum* spp. *nucleatum* has been reported to promote cancer development by activating NF-κβ signaling, altering microRNA expression, modulating autophagy, and suppressing antitumor immunity,^16^^,^^18^^,^^19^^,^^30^^,^^39^, ^40^, ^41^ and several responsible molecules, such as FadA and Fap2, have been identified as mediators through which *F*. *nucleatum* spp. *nucleatum* promotes tumor progression.^43^^,^^46^ On the contrary, it is largely unclear whether *F*. *nucleatum* spp. *animalis* exhibit similar impacts on the pathogenesis of cancers. In the present study, we revealed that *F*. *nucleatum* spp. *animalis* provokes DNA damage in EAC cells through the induction of ROS accumulation. Intriguingly, this virulence appeared to be specific to *F. nucleatum* spp. *animalis*, rather than to *F. nucleatum* spp. *nucleatum*. Although the mechanism underlying how spp. *animalis* specifically induces a greater burden of genomic damage in EAC cells compared to spp. *nucleatum* is unclear, a recent genomic analysis of *F*. *nucleatum* species has shown that *F*. *nucleatum* spp. *animalis* uniquely has genes coding ferrous iron (Fe2+) transporters.^47^ The unique iron ion dynamics of *F*. *nucleatum* spp. *animalis* may lead to the induction of genomic damage in EAC cells because ferrous iron (Fe2+) is well known to enhance the generation of free radicals. Since the genomes of the 4 spp. of *F. nucleatum* differ significantly enough to warrant reclassification into different species,^31^ it is essential to differentiate between the spp. of *F*. *nucleatum* and focus on these differences in future studies to investigate their carcinogenic involvement.

In conclusion, we demonstrated the intratumoral infection of oral pathogenic bacteria in EAC tissues and its association with immune microenvironment and tumor prognosis. Notably, the prominent presence of *F. nucleatum*, particularly spp. *animalis*, and its unique virulence regarding genomic toxicity in EAC cells underscore the significance of this bacterium. Further studies examining the differences between spp. in intratumoral bacteria and analysis using a large number of clinical samples will likely enhance our understanding of EAC pathogenesis.

## Supplementary Materials

Table A1
