# Supplementary material for: The Involvement of Oral Pathogenic Bacteria, Fusobacterium nucleatum Subspecies animalis in the Pathogenesis of Human Esophageal Adenocarcinoma
Source: Gastro Hep Adv. 2025 Mar 24;4(7):100660. doi: 10.1016/j.gastha.2025.100660 (PMC12144443; doi:10.1016/j.gastha.2025.100660)
Supplement: Table A1 [file mmc1.docx]

**Supplementary Table 1. List of primers used**

| **Target** | **Description** | **Sequence 5’-3’** |
| --- | --- | --- |
| 16S-27Fmod | Forward | TCGTCGGCAGCGTCAGATGTGTATAAGAGACAGAGRGTTTGATYMTGGCTCAG |
| 16S-338R | Reverse | GTCTCGTGGGCTCGGAGATGTGTATAAGAGACAGTGCTGCCTCCCGTAGGAGT |
| nusG region 1 primer A | Forward | CAACCATTACTTTAACTCTACCATGTTCA |
| nusG region 1 primer A | Reverse | GTTGACTTTACAGAAGGAGATTATGTAAAAATC |
| nusG region 1 primer B | Forward | TGGTGTCATTCTTCCAAAAATATCA |
| nusG region 1 primer B | Reverse | AGATCAAGAAGGACAAGTTGCTGAA |
| nusG region 2 primer | Forward | GCTTCCATTTCAAGCATAAC |
| nusG region 2 primer | Reverse | GTAACTAATATATTGGTTCCAGAAG |
| βactin | Forward | GGACTTCGAGCAAGAGATGG |
| βactin | Reverse | AGCACTGTGTTGGCGTACAG |
| IL6 | Forward | AAAGAGGCACTGGCAGAAAA |
| IL6 | Reverse | TTTCACCAGGCAAGTCTCCT |
| IL8 | Forward | AAGGAAAACTGGGTGCAGAG |
| IL8 | Reverse | ATTGCATCTGGCAACCCTAC |
| CXCL1 | Forward | TCACCCCAAGAACATCCAAA |
| CXCL1 | Reverse | TCCTAAGCGATGCTCAAACA |
| CXCL2 | Forward | GCAGGGAATTCACCTCAAGAA |
| CXCL2 | Reverse | AACACATTAGGCGCAATCCA |

**Supplementary Table 2. Age distribution by presence of oral pathogenic bacteria in EAC patients under 80 years**

|  | **Oral pathogenic bacteria** | |  |
| --- | --- | --- | --- |
|  | **Negative (n=21)** | **Positive (n=4)** | **P-value** |
| Age, years *mean* | 66.6 (48-75) | 71 (66-76) | 0.22 |

**Supplementary Table 3. Cox proportional hazards model**

|  | **Overall survival** | | **Relapse free survival** | |
| --- | --- | --- | --- | --- |
| **Variable** | **HR (95% CI)** | **P-value** | **HR (95% CI)** | **P-value** |
| Oral pathogenic bacteria | 1.51 (0.28-8.24) | 0.64 | 1.38 (0.31-6.07) | 0.67 |
| Age | 1.04 (0.96-1.13) | 0.34 | 1.04 (0.96-1.12) | 0.34 |
| pStage | 2.73 (0.97-7.71) | 0.06 | 1.65 (0.78-3.47) | 0.19 |
